# A comprehensive insight into the effect of glutamine supplementation on metabolic variables in diabetes mellitus: a systematic review

**DOI:** 10.1186/s12986-020-00503-6

**Published:** 2020-09-25

**Authors:** Hamed Jafari-Vayghan, Parisa Varshosaz, Fatemeh Hajizadeh-Sharafabad, Hamid Reza Razmi, Mahdi Amirpour, Omid Mohammad Tavakoli-Rouzbehani, Mohammad Alizadeh, Vahid Maleki

**Affiliations:** 1 Student Research Committee, Tabriz University of Medical Sciences, Tabriz, Iran; 2 Faculty of Health, Arak University of Medical Sciences, Arak, Iran; 3 Departments of Chemistry and Biochemistry, and Biology and Biomolecular Sciences Program, Laurentian University, Sudbury, ON Canada; 4 Department of Clinical Nutrition, Faculty of Nutrition and Food Science, Tabriz University of Medical Sciences, Tabriz, Iran; 5 Nutrition Research Center, Faculty of Nutrition and Food Sciences, Tabriz University of Medical Sciences, Tabriz, Iran

**Keywords:** Glutamine, Diabetes mellitus, Glycemic control, GLP-1, Oxidative stress, Systematic review

## Abstract

Diabetes mellitus is one of the most important threats to human health in the twenty-first century. The use of complementary and alternative medicine to prevent, control, and reduce the complications of diabetes mellitus is increasing at present. Glutamine amino acid is known as a functional food. The purpose of this systematic review is to determine the potential role of glutamine supplementation on metabolic variables in diabetes mellitus. For this review, PubMed, SCOPUS, Embase, ProQuest, and Google Scholar databases were searched from inception through April 2020. All clinical trial and animal studies assessing the effects of glutamine on diabetes mellitus were eligible for inclusion. 19 studies of 1482 articles met the inclusion criteria. Of the 19 studies, nine studies reported a significant increase in serum GLP-1 levels. Also, eight studies showed reducing in serum levels of fasting blood sugar, four studies reducing in postprandial blood sugar, and triglyceride after glutamine supplementation. Although glutamine resulted in a significant increase in insulin production in seven studies, the findings on Hb-A1c levels were inconclusive. In addition to, despite of the results was promising for the effects of glutamine on weight changes, oxidative stress, and inflammation, more precise clinical trials are needed to obtain more accurate results. In conclusion, glutamine supplementation could improve glycemic control and levels of incretins (such as GLP-1 and GIP) in diabetes mellitus. However, more studies are needed for future studies.

## Introduction

Diabetes mellitus is one of the major threats to human health in the twenty-first century [1]. The prevalence of this disease is rising dramatically in the world, and the global prevalence of diabetes is about 8.8% (415 million) in 2015 [2]. It is estimated that the prevalence of this disease will reach 439 million by 2030 and 642 million by 2040 [3]. Approximately 85% of patients with diabetes mellitus have Type 2 diabetes mellitus (T2DM), which can be a result of genetic predisposition, environmental factors, or a combination of these two [4].

Diabetes mellitus refers to metabolic diseases which are characterized by hyperglycemia that develops as a result of impairment in insulin secretion, insulin action, or both [5, 6]. Chronic hyperglycemia can cause macrovascular complications such as coronary artery disease, peripheral vascular disease, and cerebrovascular disease, and microvascular complications, including retinopathy, nephropathy, and neuropathy [7]. Also, chronic hyperglycemia increased inflammation and oxidative stress that play an important central role in the pathogenesis of diabetes complications [8].

Recently, the use of complementary therapy to improve and reduce the symptoms of diabetes mellitus, along with drug therapy and reduced drug dosage, have increased in use [9]. Glutamine, an α-amino acid that is used in the biosynthesis of proteins, is both non-essential and conditionally essential in humans. The body can usually synthesize sufficient amounts of glutamine, but in some instances of stress, the body's demand for glutamine increases, and glutamine must be obtained from the diet [10, 11].

Glutamine is the physiological precursor of arginine for the production of nitric oxide (NO), whose creation in β-cells potentiates insulin secretion [12]. Furthermore, glutamine creates the main source of glutamate for the production of glutathione, which is essential in reducing oxidative stress, which eventually results in maintaining inflammatory processes within β-cells in diabetes [12]. Moreover, in improving the glucose profile, L-glutamine has a positive effect on glucose oxidation and insulin resistance [13]. Oral L-glutamine enhances the circulation of gastrointestinal incretin hormones (glucagon-like peptide-1 (GLP-1) and stimulated insulin release as well as reduced (postprandial) glycemia in diabetes mellitus [14, 15].

Although several studies have shown positive effects of glutamine supplementation on metabolic variables in diabetes mellitus, there is no systematic review that summarizes the results of these studies. This study aims to evaluate the effects of glutamine on metabolic variables in diabetes Mellitus and to determine possible directions for future studies.

## Method

### Search strategy

To find relevant publications earlier than April 2020, two independent investigators performed a literature search in PubMed, SCOPUS, Embase, ProQuest, and Google Scholar electronic databases using following keywords: ““glutamine” OR “L–glutamine OR “glutamine supplementation OR “glutamine dipeptides” AND “diabetes mellitus” or “Type 2 diabetes” or “type II diabetes” or “Type 1 diabetes” or “type I diabetes” or “diabetic” or “T2DM” or “T1DM” or “noninsulin-dependent diabetes mellitus” or “insulin-dependent diabetes mellitus” or “NIDDM” or “IDDM” or “hyperglycemia” or “diabetic” or “FBS” or “fasting blood sugar” or “glycemic outcomes” or”fasting blood glucose” or “HOMA-IR” or “B-cell function” or “insulin” or “glucose” or “glycemic” or “hyperglycemic”. Reference lists and related records were manually reviewed. The search was limited to English language articles up through April 2020. This study was performed based on the Preferred Reporting Items for Systematic Reviews and Meta-Analyses (PRISMA) protocol for reporting systematic reviews and meta-analyses. The protocol for this review was registered in the PROSPERO database under registration number CRD42018090829.

### Eligibility criteria

All clinical trial and animal studies assessing the effects of glutamine on metabolic variables (e.g., glycemic status, incretin hormones, lipid profile, oxidative stress and inflammation biomarkers) in diabetes mellitus were eligible for inclusion; exclusion criteria were (1) in vitro models, and (2) studies published in non-English language journals.

### Data extraction

Data extraction was conducted independently by two investigators using a standardized data collection form. The following information was also obtained from each study: first author, year of publication, country of origin, age range, sample size, daily dose, duration of intervention, and principal outcome. The quality of the included studies was assessed by a third reviewer using primary data extraction. Finally, the reviewers discussed articles to reach an agreement.

### Quality assessment

Two independent researchers analyzed the quality of eligible studies. Randomized control trials were evaluated using Physiotherapy Evidence Database [16] (PEDro) scale, and the score are represented in Table 1.

**Table 1:** Assessment quality using the PEDro scale for randomized clinical trials

Items^a^	Chang et al.	Mansour et al.	Samocha-Bonet et al.	Takeuti et al.	Samocha-Bonet et al.	Greenfield et al.	Samocha-Bonet et al.	Lomivorotov et al.	Torres-Santiago et al.	Meek et al.
1. Eligibility criteria	N	Y	Y	N	Y	Y	Y	Y	Y	N
2. Random allocation	Y	Y	Y	N	Y	N	Y	Y	Y	Y
3. Concealed allocation	N	N	N	N	N	N	N	Y	N	N
4. Similar at baseline	Y	Y	Y	Y	Y	Y	Y	Y	Y	Y
5. Blinding subjects	Y	Y	N	N	Y	N	N	Y	Y	Y
6. Blinding therapists	N	Y	N	N	Y	N	N	Y	Y	Y
7. Blinding assessors	N	N	N	N	N	N	N	N	N	N
8. Adequate follow up	Y	Y	Y	N	Y	Y	Y	Y	Y	Y
9. Intention to treat analysis	N	N	N	N	Y	N	Y	Y	N	N
10. Between group statistical comparison	Y	Y	Y	Y	Y	Y	Y	Y	Y	Y
11. Point estimate/measure of variability	Y	Y	Y	Y	Y	Y	Y	Y	Y	Y
Score total	6/10	7/10	5/10	3/10	8/10	4/10	6/10	9/10	7/10	7/10

Y, contemplated item; N, no contemplated item

^a^Item 1 does not contribute to the total score

## Results

**Fig. 1:**
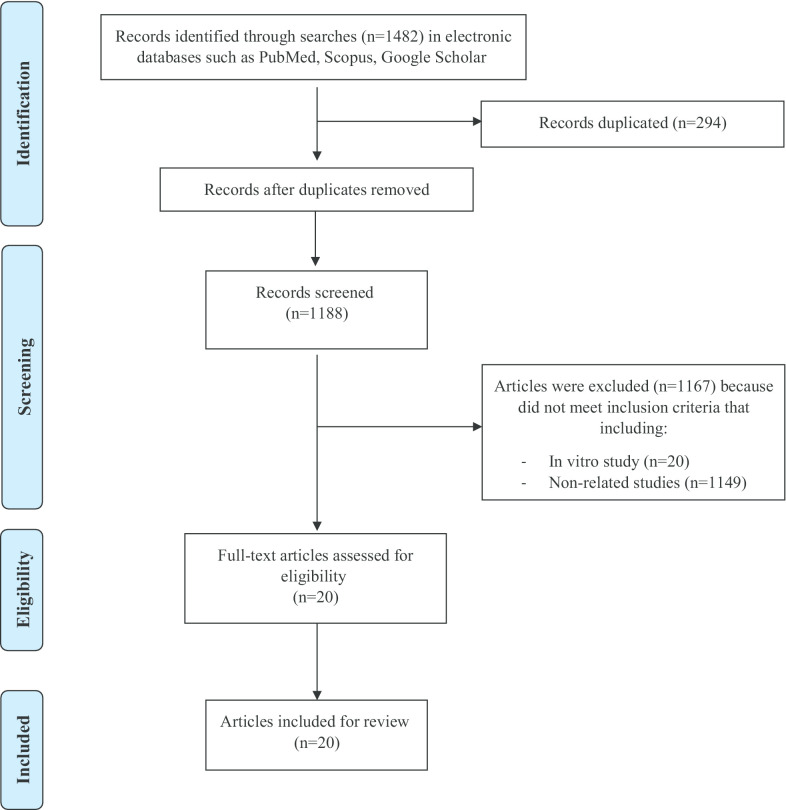
Flowchart of the process for selecting studies for the systematic review

A flow diagram of the study selection is summarized in Fig. 1. A total of 1482 articles were retrieved, of which 294 were duplicates, resulting in 1188 non-duplicated publications. Of these, 1167 articles did not meet the inclusion criteria and were excluded. Finally, 20 articles met the inclusion criteria for this review. The characteristics of the selected studies are provided in Tables 2 and 3.

**Table 2:** Characteristics of animal studies that reported the effects of glutamine on metabolic variables in diabetes mellitus

Author, year, place	Models	Daily dose	Duration	Main Outcomes
Alba-Loureiro et al., Brazil, 2009 [23]	Wistar rats were divided into four groups: 1. Control, 2. Control + glutamine, 3. Diabetic, 4. Diabetic + glutamine	1000 mg/kg	15 days	*No significant change* Fasting Blood glucose, TC, body weight gain *Significant decrease* TG *Significant increase* IL-6, IL-1
Tsai et al., Taiwan. 2011 [20]	Rats divided into three groups: 1. Normal control, 2. Diabetic fed with a common semi purified diet, 3. Identical diet with glutamine	25% of total amino acid nitrogen	6 weeks	*No significant change* Fasting Plasma glucose *Significant increase* GSH/GSSG
Tsai et al., Taiwan, 2012 [19]	Rats divided into three groups: 1. Normal control, 2. Diabetic fed with a common semi purified diet, 3. Identical diet with glutamine	25% of total amino acid nitrogen	8 weeks	*Significant decrease* GPx, SOD, *Significant increase* TAC, catalase, GRd, GSSG *No significant change* HDL, TG, LDL, TC, fasting glucose
Tsia, Taiwan, 2012 [22]	Rats divided into three groups: 1. Normal control, 2. Diabetic fed with a common semi purified diet, 3. Identical diet with glutamine	25% of total amino acid nitrogen	8 weeks	*Significant increase* GSH *Significant decrease* CRP, IL-6, IL-23, MCP-1 *No significant change* Fasting blood Glucose, TGF-β, TNF-α, IL-17A
Badole et al., India, 2013 [18]	Rats divided into six groups: 1. Nondiabetic, 2. Diabetic control, 3. Sitagliptin (5 mg/kg), 4. Glutamine 250 mg/kg, 5. Glutamine 500 mg/kg 6. Glutamine 1000 mg/kg	250–500-1000 mg/kg	8 weeks	*Significant decrease* MDA, fasting blood glucose, LDL,TC, TG *Significant increase* Plasma and pancreatic insulin, increased GLP-1, amide secretion, GSH, SOD, GPx (only the highest dose), HDL
Bdaole et al., India, 2013 [18]	Rats divided into four groups: 1. Non-diabetic, 2. Diabetic control, 3. Sitagliptin + cycloart-23-ene-3b, 25-diol (1 mg/kg) 4. Sitagliptin (5 mg/kg, p.o.) + L-glutamine (1000 mg/kg	1000 mg/kg	8 weeks	*Significant increase* SOD, GPx, GSH, insulin and GLP-1, HDL *Significant decrease* HbA1C, LDL, TG, TCF, fasting glucose
Badole et al., India, 2014 [17]	Rats divided into four groups: 1. Nondiabetic control, 2. Diabetic control (distilled water 10 mg/kg), 3. Glutamine 500 mg/kg, 4. Glutamine 1000 mg/kg	500–1000 mg/kg	4 months	*Significant decrease* fasting Plasma glucose, prevent weight loss *Significant increase* SOD and GSH *Significant decrease* MDA
Carlos Vinicius D. da Rosa et al., Brazil, 2015 [24]	Rats divided into six groups: 1. Control, 2. Diabetic + saline, 3. Control + glutamine, 4. Diabetic + glutamine, 5. Control + glutamine dipeptide (GPD), 6. Diabetic + GPD	248 mg/kg glutamine / 400 mg/kg GPD	30 days	*Significant increase* TC, HDL *Significant decrease* TG *No significant change* Fasting glucose, LDL
Bataglini et al., Brazil, 2017 [21]	Rats were divided into 8 groups: 1. Non diabetic animals, 2. Diabetic animal with no hormones or GDP, 3. Diabetic + insulin, 4. Diabetic + insulin + cortisol, 5. Diabetic + insulin + glucagon, 6. diabetic + insulin + adrenaline, 7. Diabetic + insulin + GDP, 8. Diabetic + insulin + cortisol + GDP	200–400-1000 mg /kg	0–6 min	*Significant decrease* TAC, TG, postprandial glucose, prevented weight loss
Medras et al., Egypt, 2017 [25]	Rats divided into 5 groups: 1. Vehicle group, 2. Diabetic rats receiving saline, 3. Diabetic rats receiving liraglutide, 4. Diabetic rats with glutamine, 5. Diabetic rats with liraglutide and glutamine	4.5 mg/kg	4 weeks	*Significant increase* Insulin production *Significant decrease* Fasting Blood glucose *No significant change* TC, TG

*TC* total cholesterol, *TG* triglyceride, *IL* interleukin, *GSH* glutathione, *GPx* glutathione peroxidase, *SOD* superoxide dismutase, *TAC* total antioxidant capacity, *CRP* c-reactive protein, *TGF* transforming growth factor beta, *TNF-α* tumor necrosis factor-alpha, *GLP* glucagon-like peptide, *FBS* fasting blood sugar, *HDL* high density lipoprotein, *LDL* low density lipoprotein, *MDA* malondialdehyde

**Table 3:** Characteristics of human studies that reported the effects of glutamine on metabolic variables in diabetes mellitus

Author, place, year	Type of study	Subjects	Sample size	Age (years)	Daily dose	Duration	Main outcomes
Greenfield et al., UK, 2009 [33]	RCT	T2DM	24	30–40	75 g glucose in 300 ml of water and 30 g glutamine in 300 ml of water	3 separate occasions over a period of < 1 month	*Significant increase* GLP-1, insulin production, glucagon, GIP levels
Samocha-Bonet et al., Australia, 2011 [14]	Crossovr study	T2DM	15	40–70	30 g L-glutamine (Gln-30), 15 g L-glutamine (Gln-15),100 mg sitagliptin (SIT) and 100 mg SIT plus Gln-15 (SIT + Gln-15)	1–2 weeks	*Significant reduce* postprandial glucose response *Significant increase* postprandial insulin response, C-peptide, postprandial glucagon concentration total GLP-1
Lomivorotov et al., russia, 2012 [31]	RCT	T1DM	64	Intervention: (60 ± 7)	0.4 g/kg/day of 20% solution of N(2)-L-alanyl-L-glutamine	4 weeks	*No significant change* Insulin resistance, insulin sensitivity, β-cell function, fasting blood glucose, TG
Chang et al., Australia, 2013 [29]	RCT	T2DM	20	Healthy Men: (29.5 ± 3.8) T2DM Patients: (68 ± 1.1)	7.5 or 15 g glutamine or 350 mL of 0.9% saline	0–30 min	*Significant increase* GLP-1, GIP, insulin and glucagon levels *Insignificant* Fasting blood glucose
Mansour et al., Iran, 2014 [28]	RCT	T2DM	66	18–65	30 g/d glutamine	6 weeks	*Significant reduce* Trunk fat, Total fat, Total fat free mass, HbA1c, body fat mass, percent body fat, WC, appendicular fat Fasting blood glucose *Significant increase* Trunk fat-free mass, Trunk fat mass, Appendicular fat free mass, Total fat mass, Appendicular fat, Plasma glutamine concentration, fat-free mass *Insignificant* Body weight, BMI, Fasting insulin, HOMA-IR, QUICKI, TG, Cholesterol, HDL-C, LDL-C, CRP
Samocha-Bonet et al., Australia, 2014 [26]	Crossover study	T2DM	13	40–70	glutamine (15 gbd) + sitagliptin (100 mg/d)	4 weeks	*Significant reduce* postprandial glucose *Insignificant* HbA1c, Fasting plasma glucose *Significant Increase* GLP-1,
Takeuti et al., Brazil, 2014 [30]	RCT	T2DM	11	21–60	9 g palm oil and 30 g glutamine diluted in 200 ml of water	2 separate days	*Significant reduce in palm oil group* BG and PYY “in 2 h after the stimulus”, GLP-1 “in 1 h after the stimulus” *Insignificant in palm oil group* BG and PYY “in 1 h after the stimulus”, GLP-1 “in 2 h after the stimulus” *Significant reduce in glutamine group* BG “in 2 h after the stimulus” *Insignificant in glutamine group* BG “in 1 h after the stimulus”, PYY & GLP-1 “in 1&2 h after the stimulus”
Samocha-Bonet et al., Australia, 2015 [27]	Randomized crossover study	T2DM	10	40–70	L-glutamine (25 g), protein (25 g) or water	1–2 weeks	*Significant Reduce* postprandial glycaemia *Significant increase in protein group* first-phase insulin, total GLP-1, Second-phase insulin response was significantly augmented by protein *Significant increase in glutamine group* Total GLP-1
Meek, et al. UK, 2016 [34]	Crossover study	Healthy and T2DM	37	22–30	3–6 g Ileal release glutamine	4 h	*Insignificant change* GLP-1, insulin, glucose tolerance
Torres-Santiago, USA, 2017 [32]	Crossover study	T1DM	13	8 boys and 5 girls; mean age 15.9 ± 1.6 years	Drink containing 0.25 mg/kg glutamine	4 weeks	*Significant reduce* Fasting Blood glucose *Insignificant change* Insulin sensitivity, plasma GLP-1, basFal plasma free insulin concentration

*GLP-1* Glucagon-like peptide-1, *GIP* Gastric inhibitory polypeptide, *BG* blood sugar, *PYY* peptide YY, *FBS* Fasting Blood Sugar, *TG* Triglyceride, *TC* Total Cholesterol, *HDL* high density lipoprotein, *LDL* low-density lipoprotein, *WC* waist circumference, *BMI* body mass index, *HOMA-IR* Homeostatic Model Assessment-Insulin Resistance Index, *TG*;Triglycerides, *QUICKI* Quantitative Insulin Sensitivity Check Index

### Characteristics of the included studies

In total, 20 studies were selected after meeting the inclusion criteria, including ten animal studies and nine human studies. All of the animal studies were conducted on diabetic rats, including three studies on type 1 diabetic rats and six studies on type 2 [17–25]. Besides, glutamine was supplemented with a dosage from 4.5 to 1000 mg/kg. The human studies had a wide range of glutamine dosage and supplementation duration. Six studies were conducted on diabetes type 2 patients [14, 26–30], and one was conducted on the obese and diabetic subject [15]. Also, two studies examined the glutamine effect on diabetes type 1 patients [31, 32]. Glutamine was generally supplemented by diluting in water and taken as a drink. Five human studies gave glutamine to subjects, which were over weighted or obese [14, 15, 26–28]. No oral hypoglycemic agents were taken by the subject of Greenfield et al. study [15], while others used no other hypoglycemic agents except for metformin in a stable dose [14, 28]. The characteristics of included studies are outlined in Tables 2 and 3**.**

### Glutamine, weight change in diabetes mellitus

#### Animal studies

Three studies investigated the effect of glutamine on weight change in animals. Weight in rats decreased after induction of diabetes by STZ. Although high dose supplementation of glutamine at 500 or 1000 mg/kg caused prevention of weight loss, no significant weight loss or gain was reported in these studies.

#### Human clinical trials

Obesity, especially central adiposity, is involved in diabetes pathogenesis via insulin resistance [35, 36]. Several main mechanisms were suggested by which obesity mediates insulin resistance, including altered secretion patterns of adipocytokines, increased levels of glucocorticoids in visceral fat tissue, and increased secretion of pro-inflammatory agents [35, 37]. Hence, weight loss with a focus on reducing central adiposity is one of the main priorities in management of diabetes. Mansour et al. examined supplementation of 30 g/d glutamine over 6 weeks in patients with T2DM and found that glutamine caused a significant reduction in body fat mass, percentage of body fat, and waist circumference, and a significant increase in fat-free mass, despite having no effect on overall body weight [28]. Samocha-Bonet et al. evaluated various doses of glutamine (15 g/d for 4 weeks and 25 g/d for 1–2 weeks); the results of their studies showed no significant changes in body weight [26, 27].

### Glutamine and glycemic status in diabetes mellitus

#### Animal studies

All included studies assessed the possible effect of glutamine on glycemic status in diabetic animals. However, due to the different duration of supplementation and the wide range of glutamine dosage, contradiction was observed in the results. Glutamine supplementation with three different dosages (250–500–1000 mg/kg) resulted in a significant reduction in plasma glucose level, with an increase in plasma and pancreatic levels of insulin [18]. In the most recent study conducted on this subject, 4 weeks of supplementation of 4.5 mg/kg glutamine caused a significant increase in insulin production and a decrease in glucose levels [25]. A significant reduction was observed in glucose blood level following a 4-week glutamine supplementation regime [32]. A high-dose glutamine supplementation led to postprandial glucose reduction [27]. In contrast, almost half of included studies showed no statistically significant improvement in glycemic status [19, 20, 22–24, 26, 29, 31].

#### Human clinical trials

The effects of glutamine on hormone secretion and pyloric motility seem to conform in a dose-dependent manner [36]. Mansour et al. indicated that 30 g/d glutamine for 6 weeks substantially decreased FBS and Hb A1c in patients with T2DM; however, there were no significant changes in fasting insulin and insulin sensitivity index between groups [37]. Also, oral dose of encapsulated glutamine did not stimulate consistent increase in GLP-1 and insulin secretion in type 2 diabetes patients [34]. In a study by Greenfield et al., healthy, obese subjects with T2DM or impaired glucose tolerance received oral glucose (75 g), glutamine (30 g).

In another study by Samocha-Bonet et al., T2DM patients consumed 15 g glutamine with 100 mg/d sitagliptin or 15 g glutamine with a placebo [38]. After 4 weeks, L-glutamine decreased HbA1c and fructosamine irrespective of sitagliptin in patients with T2DM, and postprandial glucose decreased with significant time-treatment interactions, while HbA1c and fructosamine decreased without significant time-treatment interactions [38]. Samocha-Bonet et al. reported reduced early postprandial glycemia in patients with T2DM after administration of a single dose of 30 g of glutamine or 15 g glutamine with or without sitagliptin [39]. Glutamine at both dosages significantly increased postprandial insulin response and glucagon levels; however, C-peptide levels were not affected [28]. In contrast, 4 weeks of glutamine supplementation (0.4 g/kg) did not show any significant changes in insulin resistance, insulin sensitivity, β-cell function, or blood glucose in patients with T1DM [31]. Also, administering 0.25 g/kg glutamine to adolescents did not change glycemic parameters such as insulin sensitivity or basal plasma free insulin concentration, except for blood glucose after 4 weeks in patients with T1DM [32]. This may, however, be due to the low dose of glutamine.

### Glutamine and incretin hormones in diabetes mellitus

#### Animal studies

Incretin hormones trigger physiological pathways to release insulin following a meal [38]. Some evidence suggests hyperglycemic state decreases GLP-1 secretion in T2DM [39, 40]. Glutamine may stimulate GLP-1–secreting cells to release GLP-1 [41]. Two of the studies in which incretin hormones were evaluated showed a significant increase in GLP-1 after 8 weeks of high-dose glutamine supplementation.

#### Human clinical trials

Glutamine significantly increased GLP-1 compared to water alone. Elevation of circulating GLP-1 levels following the ingestion of glutamine was detectable as early as 15 min post-ingestion. Glutamine also caused a significant increase in plasma insulin levels, particularly in obese subjects; however, glucagon levels also increased. GIP levels increased following glutamine consumption; however, the effectiveness was less than that of glucose [35].

The mechanism by which glutamine influences glycemic status may be explained by GLP-1–induced slowing of gastric emptying [28]. Chang et al. evaluated intraduodenal (ID) infusions of glutamine (7.5 or 15 g) or saline over 30 min in healthy subjects and 15 g glutamine or saline in T2DM patients followed by an ID infusion of glucose over 100 min [36]. The results showed that 15 g ID glutamine significantly enhanced GLP-1 and glucagon concentrations with modest increments in insulin levels and phasic pyloric pressures in both groups [36]. Glutamine infusion significantly stimulated GIP only in T2DM patients, while the glucose load was not decreased, likely due to elevated levels of glucagon [36].

The changes of active GLP-1 and insulin-to-glucose AUC were also incremental with a significant time-treatment effect; however, fasting total GLP-1, fasting active GLP-1, and postprandial total GLP-1 increased without a significant time-treatment interaction [38]. In a study by Takeuti et al., T2DM patients consumed 30 g of glutamine diluted in 200 ml of water for one day, and the authors observed a significant reduction in blood glucose 2 h after the ingestion of glutamine; however, blood glucose 1 h afterward and PYY and GLP-1, 1 and 2 h after glutamine consumption showed no significant changes. Samocha-Bonet also examined the effect of oral L-glutamine [25 g], whole protein low in glutamine (25 g), or water on the concentration of incretin hormones and insulin response in well-controlled T2DM patients and found that the first-phase insulin response and total GLP-1 were enhanced following the ingestion of both L-glutamine and protein; however, only protein potentiated the second-phase insulin response [27].

### Glutamine and lipid profile in diabetes mellitus

#### Animal studies

Among the included studies, six examined the possible effect of glutamine on lipid parameters. Eight weeks of administering 1gr/kg glutamine resulted in a significant reduction in LDL, TC, and TG levels, and a significant improvement in HDL levels [18]. Three other studies showed a reduction in TG levels after glutamine supplementation; however, no statistically meaningful change was observed on other lipid parameters, including LDL, TC, and HDL [21, 23, 24]. In addition, Tsai et al. found no significant changes after glutamine supplementation on the lipid profile [19]. Although most included studies support the useful effects of glutamine on TG levels, it is premature to reach a specific conclusion for other parameters.

#### Human clinical trials

Only two human studies evaluated the effect of glutamine on the lipid profile, and neither resulted in a significant change. 30 g/day for 6 weeks showed no significant changes in LDL, HDL, TG, or TC levels [28].

### Glutamine and oxidative stress and inflammation biomarkers in diabetes mellitus

### Animal studies

Seven studies evaluated the effects of glutamine on oxidative stress and inflammatory biomarkers. Glutamine dosage was between 250 to 1000 mg/kg among the studies, and supplementation duration was from 15 days to 4 months. Three studies conducted by Badole et al. showed a significant reduction in oxidative stress, which resulted from an improvement in SOD, MDA, GSH, TAC, and CAT after supplementation with three different dosages (250–500–1000 mg/kg) of glutamine for over two months [17, 18]. In another study, 1000 mg/kg of glutamine showed a significant increase in IL-1 and IL-6 levels after 15 days. Tsia et al. found that supplementing glutamine for 6–8 weeks led to a reduction of CRP, IL-23, IL-6, and MCP-1 levels. However, no changes were observed in TGF-β, TNF-α, or IL-17A [19, 20, 22].

#### Human clinical trials

In the only human study that evaluated the effect of glutamine of inflammatory markers, Mansour et al. found no significant change in CRP after supplementing 30 g/day glutamine for 6 weeks [28].

## Discussion

The results of this systematic review showed that glutamine supplementation has a potential effect on improving fasting plasma glucose [17, 18, 22, 25, 28, 29, 31, 32], postprandial blood glucose [14, 21, 26, 27, 30], and significant increases in insulin production and incretin hormones such as GIP and GLP-1 [18, 26, 27, 29, 30, 33]. However, results from insulin sensitivity were contradictory [31, 32]. Regarding HbA1c and HOMA-IR, there is a lack of sufficient evidence to reach any conclusion.

Generally, incretin hormones such as GLP-1 and GIP are released from intestinal L-cells and play an essential role in physiologically mediating insulin secretion after a meal [14]. Since GLP-1 production is assumed to remain intact in well-controlled diabetic patients and stimulates insulin release and lowers postprandial glycemia, several therapeutic approaches are being developed to increase GLP-1 action for treating diabetes mellitus [27]. Glutamine, a nonessential amino acid, is the most common free amino acid found in body fluids and skeletal muscles and has a pivotal role in regulating cell proliferation and growth [30] as well as stimulating incretin hormones, particularly GLP-1 secretion [15]. Interestingly, significant reductions in glutamine concentration have been found in diabetes mellitus compared with healthy individuals [28]. Nevertheless, studies have shown significant increases in GLP-1 levels following additional glutamine administration in diabetes mellitus [26, 27, 42].

Different mechanisms for glutamine signaling pathways should be taken into account. First, glutamine uptake with GLUTage cells is sodium-dependent, which could itself initiate GLP-1 secretion [33]. Indeed, in vitro and epidemiological studies have demonstrated elevating effects of glutamine on GLP-1 secretion in GLUTage cells more than other amino acids and even glucose [15, 41]. In this context, one in vitro study has found that glutamine causes membrane depolarization initiation and, subsequently, calcium entry into cells, which ultimately leads to GLP-1 secretion [14]. In addition, the effects of glutamine on lowering glucose levels could be due to two different GLP-1-dependent mechanisms, including possibly stimulating insulin secretion or, more likely, slowing the gastric emptying rate [29]. In this regard, in healthy subjects, having a mixed meal, a combination of protein, carbohydrates, and fat, followed by a higher energy expenditure, prolonged the rate of gastric emptying and resulted in lower glycemia compared with a meal of carbohydrates alone [14]. Additionally, GLP-1 regulates insulin secretion from pancreas β-cells in both normal and disease conditions [27]. Likewise, glutamine, through GLP-1 mediation and in a dose-dependent manner, increases insulin release in diabetes mellitus [29]. An in vivo study on a high fat diet enriched with glutamine administered to Wistar rats found that glucose uptake increased with stimulation of insulin signaling in skeletal muscles and reduced hepatic gluconeogenesis, which both showed improvement in insulin sensitivity [43].

Dyslipidemia is one of the major complications of diabetes mellitus; thus, reducing lipid profile parameters may play a protective role in cardiovascular disease. Results demonstrated significant reductions in TG levels after glutamine administration [18, 21, 24]. However, the findings on TC, LDL, and HDL were contradictory [18, 19, 23, 24, 28]. Additionally, no significant change was observed in human studies, and the results were insufficient to draw any conclusions [28]. Hyperglycemia causes a significant increase in lipid profile levels, which may be related to a lack of insulin. The normalization of glycemic status is shown to have a significant effect on the lipid profile. Glutamine could have a lipid-lowering effect by increasing GLP-1 secretion. Increased levels of GLP-1 are associated with a reduction in lipid absorption [18]; in addition, it appears that GLP-1 can directly reduce hepatic lipogenesis and expression of lipogenesis-related genes through the cAMP/AMPK pathway [44]. In another study, GLP-1 was able to decrease lipid accumulation in the absence of insulin [45].

Anthropometric data derived from the studies is inadequate, although glutamine prevented weight loss after diabetes induction in animals [17, 21]. Although trunk fat, total fat, and total fat-free mass were all significantly decreased, no significant changes were observed in BMI or body weight in human studies[28]. Obesity is a well-known modifiable risk factor for diabetes that can be managed by nutritional therapy [33]. It has been suggested that additional glutamine intake has anti-obesity as well as antidiabetic properties [46]. A hypothesized mechanism is attributed to L-cells that co-produce GLP-1 and GLP-2 at the same time, which regenerates intestinal epithelium, mediates peptide YY production, and subsequently, through appetite suppression, prolongs satiety through GLP-1 receptors and thus manages weight control [33, 47].

Changes in body composition are a common feature in diabetic patients with abnormal decreases in lean body mass, particularly in elderly individuals [28]. Concerning this deterioration, in vivo evidence has demonstrated the protective role of glutamine on reducing fat mass (FM) and waist circumference (WC) as well as increasing fat-free mass (FFM) without significant changes in body weight [28]. It is possible that glutamine, by increasing GLP-1 levels, which mediate FM and body weight reduction or replace FM with muscle, improves body composition in diabetes mellitus [28]. GLP-1 also affects adipose tissue, leading to increased lipolysis and thermogenesis in brown adipose tissue [48, 49]. Given the beneficial effects of glutamine on GLP-1, it is recommended to conduct clinical trials on the effects of glutamine on the expression of the genes involved in lipogenesis and thermogenesis in fat tissues. Studies have shown that GLP-1 leads to the suppression of the appetite center in the central nervous system, and a decrease in the secretion of the ghrelin hormone and gastric emptying [50, 51]. On the other hand, glutamine can lead to an increase in serum levels of GLP-1. Therefore, it is recommended the effect of glutamine on hormones involved in appetite, especially ghrelin, should be considered in future directions.

**Fig. 2:**
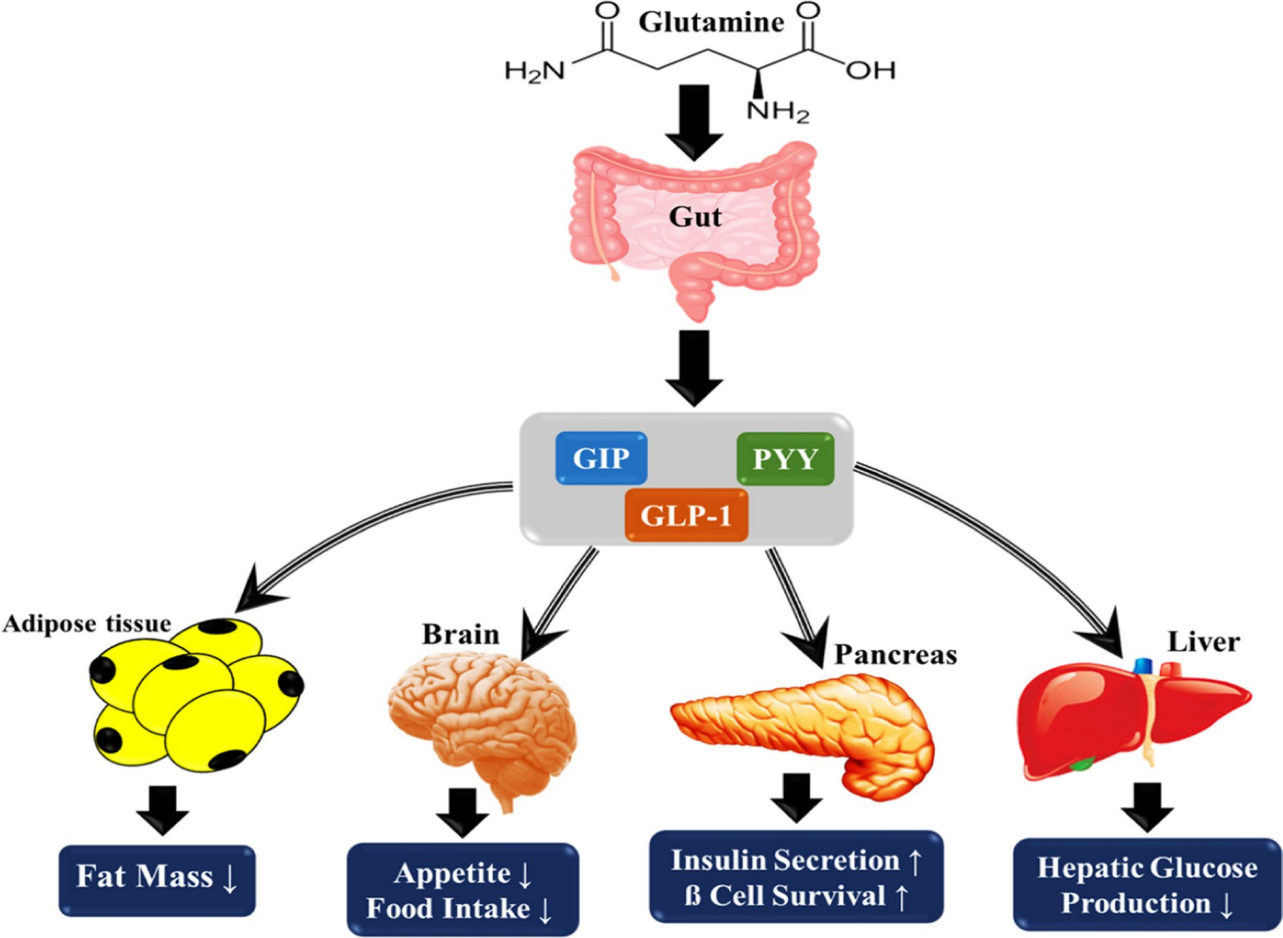
Potential roles of glutamine supplementation effects on metabolic variables in diabetes mellitus

Results suggest a potential effect of glutamine on oxidative stress and inflammatory markers. Glutamine supplementation showed a significant increase in SOD, GSH, GPx, and catalase in animal studies [17–20, 22] as well as meaningful alleviations in CRP, IL-6, IL-23, and MCP-1 levels [22]. Glutamine may have an antioxidant effect due to its role in glutathione synthesis. It can increase the enzyme activity of glutathione peroxidase and reduce ROS production [17, 18]. Thus, it can increase the total antioxidant level and activity of SOD and catalase enzymes. It is similarly indicated that oxidative stress may lead to inflammation through an increase in gene expression of NF-κB and inflammatory biomarkers [52, 53]. Since oxidative stress and inflammation play a vital role in pathogenesis and side effects of diabetes, glutamine may help improve the glycemic status and ameliorate side effects of diabetes, due to its antioxidant and anti-inflammatory effects [54]. Overall, possible and potential roles of glutamine on the metabolic state in diabetes mellitus are shown in Fig. 2.

## Conclusion

This systematic review found that glutamine supplementation can lead to a decrease in fasting blood glucose, post-meal glucose, and triglyceride levels and an increase in insulin production. However, the results on the effect of glutamine on Hb-A1c and TC, LDL, and HDL levels were inconclusive. Glutamine supplementation also resulted in increased levels of GLP-1.

Although the outcomes seem promising for the effects of glutamine on weight changes, oxidative stress, and inflammation, more precise clinical trials are needed to obtain more accurate results.

## Data Availability

Not applicable.
